# Stroke knowledge among diabetics: a cross-sectional study on the influence of age, gender, education, and migration status

**DOI:** 10.1186/1471-2377-13-202

**Published:** 2013-12-14

**Authors:** Birgitta M Weltermann, Youcef Driouach-Bleckmann, Sabrina Reinders, Peter Berndt, Stefan Gesenhues

**Affiliations:** 1 Institute for General Medicine, University of Duisburg-Essen, Hufelandstr 55, 45122 Essen, Germany

**Keywords:** Health education, Stroke knowledge, Diabetes, Migrants’ health

## Abstract

**Background:**

Stroke campaigns are educating about the need to immediately contact the emergency medical system if symptoms occur. Despite higher stroke rates among patients with diabetics and some migrant populations, there are few data about stroke knowledge in these groups.

**Methods:**

We performed a cross-sectional questionnaire survey among 250 diabetes patients from Germany and Turkey in a primary care and diabetes practice center. The two-page questionnaire asked for stroke knowledge and socio-demographic data. Also, medical and communication data were obtained. Stroke knowledge was defined as good if a participant knew (1) at least two stroke symptoms (good symptom knowledge) and (2) that immediate hospital admission or an emergency call is necessary in case of stroke symptoms (good action knowledge).

**Results:**

A total of 231 of 250 patients took part in the survey (participation rate 92.4%) with 134 natives (53.6%), 84 migrants from Turkey (33.6%) and 13 migrants (5.2%) from other countries. Comparing natives and migrants from Turkey good symptom knowledge was documented in 52.8% of the participants, good action knowledge in 67.9%, and good stroke knowledge in nearly forty percent (39.4%) of patients (n = 218). A logistic regression analysis showed better stroke knowledge if patients were younger than 61 years, had good language abilities and were living in an one-generation household (p < 0.05), while gender, years since migration and diabetes control did not play a role.

**Conclusions:**

We documented stroke knowledge deficits among patients with diabetes, both natives and migrants. Additional information strategies for these high risk populations are needed.

## Background

Higher stroke rates are well documented in diabetes patients and in various migrant populations [1-6]. The relative stroke risk is increased by 1.8 to 6-fold in patients with diabetes [1]. Data from the US Bureau of Census documented a 1.3 times higher stroke-related mortality among foreign-born than locally born females [3]. Official Dutch mortality data of the years 1995 – 2000 showed a 1.4 times higher mortality rate from cerebrovascular diseases among Turkish male migrants compared Dutch men while inequalities among females were small [7].

Despite this higher risk, there is little information about stroke knowledge among diabetics, and especially migrants suffering from diabetes. Stroke information campaigns are educating the public about the need to immediately contact the emergency medical system when symptoms occur [8,9]. However, migrants may not be reached by these strategies because of language or cultural barriers [10]. Because migrants from Turkey are among the largest ethnic minority groups in Europe [11], we were interested to understand if additional information strategies are needed for these migrant patients with diabetes.

Using a cross-sectional design we compared stroke knowledge among diabetics from Germany and Turkey, both living in the same neighborhood in an industrialized area in Germany.

## Methods

### Study population and sampling strategy

This cross-sectional study was performed among type 2 diabetes patients of a primary care and diabetes practice center located in the Ruhr area, Germany. This region is a heavily industrialized, steal producing and former coal mining region which used to attract foreign workers. The scenario was chosen for the survey because it is a combined family and diabetes teaching facility, and is providing care to patients in a region with a high proportion of migrants from Turkey^a^. All adult family practice patients with type 2 diabetes from Germany and Turkey treated between November, 2011 and May, 2012 were asked to participate in this study and if they wanted to fill the questionnaire in German or Turkish.

### Survey instrument

A two page, self-applicable questionnaire addressed stroke knowledge. Using three open questions, participants were asked to list stroke symptoms, risk factors, and the body part affected in a stroke. In three closed questions, appropriate actions in case of stroke symptoms and the critical 3-hour time interval were addressed. According to our definition, a participant had good stroke knowledge if the two following criteria were met: (1) participant knew at least two stroke symptoms (good symptom knowledge), and (2) participant knew that immediate hospital admission or an emergency call is necessary in case of stroke (good action knowledge). These criteria were identical to those used in our two prior surveys to allow comparison [12].

The questionnaire had been developed from an instrument used by Kothari and coworkers from the University of Cincinnati to study patients’ stroke knowledge [8]. It was used in three prior studies addressing stroke knowledge among the working population (German PROCAM study), seniors (German Augsburg Senior Citizen Study) as well as stroke support group members [12,13]. The German questionnaire had been translated into Turkish by two bilingual professionals. Later, it was translated back by a second native Turkish speaker, and the versions were found compatible by the research team. Content validity and cultural acceptance were evaluated separately by two bilingual medical assistants. Face validity was studied by four bilingual Turkish migrants who were asked to assess the questionnaire and indicated if they felt ambiguity or difficulties in responding to the Turkish version of the stroke questionnaire.

Medical data were retrieved from the electronic medical records: age, gender, current diabetes therapy, duration of diabetes, which diabetes complications are known, last HbA1c, prior myocardial infarction or coronary artery bypass surgery, stroke risk factors, and if the patient had a stroke. Additionally, health service data were obtained: participation in a disease management program, number of years in the practice, and number of practice contacts in the last year.

Physicians rated if the patients’ German language was sufficient for the medical consultation or if a translator was required. Additionally the following socio-demographic data were requested: marital status, health insurance, education, employment status, current living situation, number of persons and generations in the household, country of origin, number of years since migration, if the patient was a first, second or third generation migrant. After filling the questionnaire, each participant received a stroke information leaflet in German or Turkish, as wished, and additional verbal physician information about stroke warning signs and what to do in case of symptoms.

### Statistical analysis and ethical statement

The statistical analysis was performed with the IBM SPSS software package version 20.0. Comparisons of categorical data were based on the Chi-Square test. If subgroups contained fewer than 5 counts per group, Fisher’s Exact test was used for analysis. Wilcoxon test was performed for ordinal variables; continuous data were compared using the T-test statistics. Univariate analyses were performed to detect associations between the three stroke knowledge parameters and various independent parameters: age, gender, education, language abilities, health service parameters, current professional and living situation, medical data, and migration characteristics. Variables reaching statistical significance in the univariate analysis were included in the final multivariate logistic regression model. The logistic regression analysis was used to calculate odds ratios of various socio-demographic indicators for good stroke knowledge. Statistical significance was assigned at the level of p < 0.05. - The study was approved by the Ethics Committee of the University Clinic Essen, University of Duisburg-Essen, Germany. All participants gave written informed consent for the survey.

## Results

### Survey data

The participation rate was 92.4% (231 of 250 patients). Thirteen of the 231 questionnaires were not included in the analysis because these patients had migrated from other regions. According to the original study design, the subsequent analyses were performed comparing diabetics from Germany and Turkey (n = 218). All German patients were type 2 diabetics (n = 134), while the subgroup with patients from Turkey (n = 84) included three patients with type 1 diabetes incidentally. Because stroke risk is high independent of the type of diabetes, we decided to include these patients in the final analysis. Fifty-one percent of the migrants from Turkey were first generation migrants (51.2%, n = 43), while the others were second or third generation migrants (48.8%, n = 41).

### Medical and socio-demographic characteristics of the study population

Comparing the native and migrants subgroups there were no significant differences with regard to gender, insurance in a sickness fund and current or last professional status as blue collar worker. In average, the native patients were twelve years older. A higher percentage of natives were retired (66.4% vs. 28.6%, p < 0.001) and had at least eight years of education (100% vs. 64.3%). Migrant patients lived in households with more persons (median 3.5 vs. 2.0, p < 0.001) and generations (median 2 vs. 1, p < 0.001). For details see Table 1.

**Table 1:** Sociodemographic characteristics of the participants (n = 218)

	**Natives (n = 134)**		**Migrants (n = 84)**		
	**n**	**%**	**n**	**%**	***p*-value**
**Males**	65	48.5	45	53.6	0.467
**Married**	86	64.2	77	91.7	<0.001
**Retired**	89	66.4	24	28.6	<0.001
**Eight or more years of education**	134	100	54	64.3	<0.001
**Sickness fund**	131	97.8	84	100	0.286
**Blue collar worker**	22	16.4	17	20.2	0.474
**Filled questionnaire in Turkish**	0	0.0	64	76.2	<0.001
**Filled questionnaire in German**	134	100	20	23.8	<0.001
**Good language abilities**	133	99.3	25	29.8	<0.001
**Needs language support for medical care**	1	0.7	59	70.2	<0.001
**Age in years, *mean (span)***	66 (34–89)		54 (31–79)		<0.001
**Number of children in household, median, (IQR; span)**	2 (1; 0–5)		3 (1; 0–8)		<0.001
**Persons in household, median (IQR; span)**	2 (1; 1–7)		3 (3; 1–10)		<0.001
**Generations in household, median (IQR; span)**	1 (0; 1–3)		2 (1; 1–3)		<0.001
**Years since migration, average (span)**	______		37 (14–48)		--

In the final study population (n = 218), diabetes was diagnosed about 7.8 years prior in average (standard deviation 5.6 years, span 1 – 32 years). The frequency of various diabetes complications was significantly higher among the natives (48.5% vs. 31%, p = 0.01) which may be attributed to the fact that they were more than ten years older in average. Diabetes therapy was dietary only in 33.5% of the patients, metformin in 56.0%, and insulin therapy in 22.9%. For details see Table 2.

**Table 2:** Medical characteristics of the study populations (n = 218) (% unless marked otherwise)

	**Natives**		**Migrants**		
	**N**	**%**	**n**	**%**	**p-value**
**Diabetes type II**	134	100	81	96.4	0.056
**Diabetes-related diseases**	65	48.5	26	31.0	0.011
Polyneuropathy	58	43.3	25	29.8	0.045
Carotid artery disease	28	20.9	18	21.4	0.925
Nephropathy	19	14.2	10	11.9	0.630
Retinopathy	10	7.5	4	4.8	0.574
Diabetic feet syndrome	9	6.7	2	2.4	0.210
Renal insufficiency	6	4.5	9	10.7	0.077
Coronary disease	37	27.6	12	14.3	0.022
Past myocardial infarction	20	14.9	10	11.9	0.529
Coronary artery bypass	8	6.0	3	3.6	0.536
Peripheral vascular disease	13	9.7	7	8.3	0.733
**Other stroke risk factors**					
Hypertension	124	92.5	63	75.0	<0.001
Atrial fibrillation	10	7.5	2	2.4	0.135
Hyperlipidemia	71	53.0	36	42.9	0.145
Current smoker	24	17.9	23	27.4	0.098
Former smoker	6	4.5	16	19.0	0.001
Obesity with BMI ≥30	73	54.5	53	63.1	0.210
Regular alcohol intake	4	3.0	0	0.0	0.301
Prior stroke	18	13.4	9	10.7	0.553
Oral contraceptive	1	0.7	6	7.1	0.014
Family history of stroke	17	12.7	22	26.2	0.011
**Diabetes therapy**					
Participant in disease management program	134	100	84	100	
Dietary therapy only	47	35.1	26	31.0	0.530
Metformin	72	53.7	50	59.5	0.402
Insulin therapy	31	23.1	19	22.6	0.930
Years in practice, average (span), *yrs*	13.5 (1–36)		19.0 (1–40)		<0.001
Practice contacts in last year, average (span), *n*	10.4 (1–21)		9.4 (1–23)		0.109
HbA1c, average (SD),%	6.9 (5.0-13.7)		7.3 (5.4-12.4)		0.319
LDL, average (SD), *mg/dl*	128 (37–260)		122 (32–233)		0.098
Blood pressure, average, *mmHg*	140/79		136/80		0.409

Additional stroke education was of interest to 88.1% of the population. Using a multi-select question format the following educational modes were preferred: 66.1% by physician (n = 144), 24.8% by brochure (n = 54), 18.8% television (n = 41), 7.3% newspaper (n = 16). The migrants were more interested in information by their physician (72.6%) and television (21.4%), while the natives preferred information by their physician (61.9%) or a brochure (33.6%). For details see Table 3.

**Table 3:** Stroke knowledge (n = 218)

	**Natives (n = 134)**		**Migrants (n = 84)**		
	**n**	**%**	**n**	**%**	**p-value**
**Stroke symptoms**					
Motor	65	48.5	31	36.9	0.093
Language or speech	49	36.6	6	7.1	<0.001
General symptoms	37	27.6	14	16.7	0.063
Vision	15	11.2	5	6.0	0.192
Sensory	8	6.0	25	29.8	<0.001
Cranial nerve	33	24.6	11	13.1	0.039
Coma	11	8.2	4	4.8	0.416
**Stroke risk factors recalled**					
Hypertension	48	35.8	12	14.3	0.001
Smoking	47	35.1	18	21.4	0.032
Hyperlipidemia	9	6.7	1	1.2	0.093
Diabetes mellitus	24	17.9	13	15.5	0.641
Alcohol	21	15.7	11	13.1	0.601
Heart disease	5	3.7	4	4.8	0.736
**Whom would you contact in case of a stroke?**					
Emergency medical system (911)	76	56.7	22	26.2	<0.001
**What is recommended in case of stroke?**					
Rapid hospital admission, even when symptoms improve	88	65.7	42	50.0	0.014
**When to start therapy**					
First 3 hours	76	56.7	25	29.8	<0.001
**Personal experience with stroke**					
Had stroke	18	13.4	9	10.7	0.553
Have risk for stroke	65	48.5	24	28.6	0.004
Doctor says my stroke risk is high	50	37.3	8	9.5	<0.001
**Interest in stroke information**					
Wishes additional information	115	85.8	77	91.7	0.195
By TV	23	17.2	18	21.4	0.433
By newspaper	11	8.2	5	6.0	0.534
By brochure	45	33.6	9	10.7	<0.001
By physician	83	61.9	61	72.6	0.105
**Stroke knowledge summary indicators**					
Good symptom knowledge	82	61.2	33	39.3	0.002
Good action knowledge	101	75.4	47	56.0	0.003
Good stroke knowledge	67	50.0	19	22.6	<0.001

### Stroke knowledge

When asked for the body part affected by a stroke 50% of the native diabetics (67 of 134) and 25% of the migrant diabetics (21 of 84) listed the brain or head (p < 0.001). More than forty percent of the population (46.3%) knew that stroke therapy is best started within three hours but there was a significant difference according to migration status (natives 56.7% vs. migrants 29.8%, p < 0.001). Nearly sixty percent (59.6%) would recommend a rapid hospital admission, even when symptoms improve (natives 65.7% vs. migrants 50.0%, p = 0.014), and 45% would contact the emergency medical system (911) in case of a stroke (natives 56.7% vs. migrants 26.2%, p < 0.001).

The median for stroke symptoms known was 2 (IQR = 1): 24.3% did not know any stroke symptom, whereas 22.9% recalled one, 28.9% two, 18.3% three, and 5.5% named four or more symptoms.

Regarding stroke knowledge indicators, approximately 53% of the diabetics had good symptom knowledge (52.8%) and 67.9% had good action knowledge. Good stroke knowledge was demonstrated by 39.4% of the participants.

The stroke symptom knowledge was influenced significantly by one-generation household (59.1% vs. 42.0%, p = 0.014), good language abilities (58.2% vs. 38.3%, p < 0.01), self-assessment of risk for stroke (64.0% vs. 44.1%, p < 0.01), at least nine years of education (81.1% vs. 47.0%, p < 0.01), German parentage (61.2% vs. 39.3%, p < 0.01), having not more than two children (60.0% vs. 43.0%, p = 0.013). The median for stroke symptoms named within the migrant group was 1 (IQR = 2) and for natives 2 (IQR = 2). Similarly, the median for number of risk factors listed was 1 (IQR = 2). No risk factor was recalled by 33.9%, while one risk factor was named by 23.9%, two by 20.2%, three by 16.1%, and four or more risk factors by 5.9% of the participants.

Good action knowledge was influenced by one-generation household (74.5% vs. 56.8%, p < 0.01), having a coronary artery disease (79.6% vs. 64.5%, p < 0.05), good language abilities (73.4% vs. 53.3%, p < 0.01), immigrated in second or third generation or being a native (71.4% vs. 53.5%, p = 0.024), being a native (75.4% vs. 56.0%, p < 0.01), answering the questionnaire in German (73.4% vs. 54.7%, p < 0.01), and not having more than two children (78.4% vs. 53.8%, p < 0.01).

**Figure 1:**
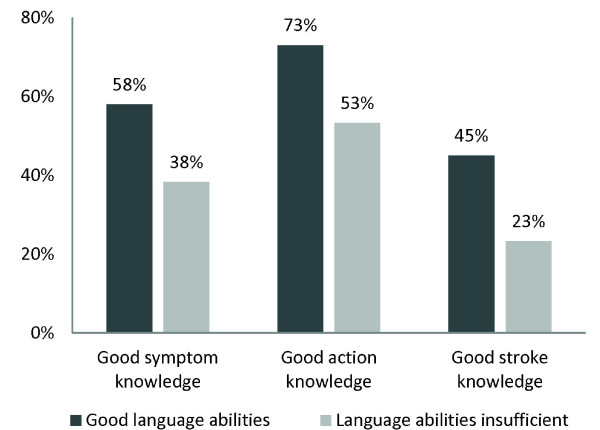
Comparison of three stroke knowledge indicators stratified according to language abilities (n = 218): the differences between subgroups were significant for all three indicators (p < 0.05).

Factors influencing good stroke knowledge significantly were one-generation household (48.2% vs. 24.7%, p < 0.01), self-assessment of risk for stroke (47.2% vs. 33.9%, p = 0.05), at least nine years of education (70.3% vs. 33.1%, p < 0.01), being a native (50.0% vs. 22.6%, p < 0.01), answering the questionnaire in German (44.8% vs. 26.6%, p = 0.012), not having more than two children (50.4% vs. 24.7%, p < 0.01), and good language abilities (45.6% vs. 23.3%, p < 0.01). For all three stroke knowledge indicators, good language abilities were a significant positive predictor. For details see Table 3 and Figure 1.

### Factors influencing stroke knowledge

Multivariate logistic regression analysis showed that good stroke knowledge was significantly associated with age, good language abilities and one-generation household, while gender, years since migration and diabetes control did not play a role. Country of origin did not play a role if controlled for good language abilities. Detailed results are listed in Table 4.

**Table 4:** Multivariate analysis: significant predictors for good stroke knowledge

	**OR**	**95% CI**	***p*-value**
**Age < = 60 years**	2.88	1.49 - 5.56	0.002
**Good language abilities**	2.26	1.09 - 4.68	0.028
**One generation in household**	3.73	1.82 - 7.65	0.0001

## Discussion

### Stroke knowledge among high risk patients and influence of migration status

Our study addressed stroke knowledge among patients with diabetes: only 39.4% of the patients knew at least two stroke symptoms and to seek immediate professional help in case of symptoms. Patients from German origin and migrants with good communication abilities showed significant better stroke knowledge than migrants who needed translation for their medical care despite the questionnaire offered in their first language.

During the last two decades, insufficient stroke knowledge has been documented in various populations [8,12,14]. In 1997, Kothari published that 39% of US stroke victims did not know any stroke symptom or sign [8]. Our study fifteen years later in a different scenario showed a slightly better knowledge only, yet 24% of high risk individuals still did not know any stroke symptom. Data from the 2003–2005 Behavioral Risk Factor Surveillance Survey showed lower stroke knowledge scores among US midlife women with Hispanic or African-American background, less than a high school education, a low annual household income and those not having a health insurance [15]. Focusing only on Hispanic males as high risk group the same survey demonstrated marked within-group disparities: heart attack and stroke knowledge was less among participants without high school education, uninsured, and those who had deferred medical care because of cost [16]. Our results are in agreement with these observations: patients with less education, less language abilities and those being first generation migrants had less stroke knowledge. In line with other studies about health knowledge and health disparities among migrants and minorities [10,17] our study suggests that special information strategies are needed for high risk individuals with migrant background.

### Diabetes, stroke risk and migration

The excess relative stroke risk due to diabetes is estimated as 1.8 to 6.0 fold [1]. Increased diabetes and stroke risks were documented in various migrant populations [2-6]. Yet, when talking about these populations, it is important to realize that the term “migrants” refers to an extremely heterogeneous group of persons [18], spanning e.g. from well-trained engineers to untrained blue collar workers migrating to Western nations. Pooled data from seven European countries published in 2012 showed that the relative risk of diabetes-related mortality was 1.9 times higher in migrant males and 2.2 times higher in migrant females compared to local-borns [19]. There was an inverse relationship between GDP (gross domestic product) of country of birth and diabetes mortality rates: the highest rates were documented in migrants from low GDP per capita countries, especially those from the Caribbean, North Africa and South Asia [19]. Etiologically, these increased stroke rates are attributed to changes in nutrition habits and life-style factors [20], however further research and the search for novel risk factors in various migrant populations are needed [21].

### Stroke knowledge, health education and migrant populations as target groups

Medical anthropology is teaching that health related beliefs and behaviors are influenced by a variety of factors such as individual, educational, socioeconomic, environmental and cultural factors, all of which interrelate [22]. In various countries and scenarios public campaigns using mass media such as television and radio, public events, information leaflets were shown to positively affect public stroke knowledge [14]. However, many studies evaluated only the short-term effects of such interventions and their long-term benefit remains unknown [14]. The SWIFT-Study (Stroke Warning Information and Faster Treatment study) is using culturally tailed interventions to improve stroke knowledge which include bilingual materials as well as integration of community resources [23]. Qualitative and quantitative studies have stressed the importance to address communities with high risk individuals. In the US hip hop stroke project a three hour music teaching session successfully educated children from high risk communities about stroke symptoms and how to react in case of stroke symptoms [24]. The educational effect of this community intervention persisted after 15 months [9]. Also, children were an effective knowledge conduit for their parents: parental stroke literacy defined as knowing five symptoms and to call the ambulance increased from 3.9 to 29.6% [25]. Qualitative anthropological research using interviews of diabetes patients, their families and care providers in a Berlin Turkish community showed that diabetes is a “social experience” and “family affair”, especially in patients living in traditional more generation families [26]. We showed that first generation migrants with poor language abilities living in more generation families showed knowledge deficits, yet it is likely that the family’s knowledge about stroke is better and may constitute a protective factor: if a senior in a more generation family is showing stroke symptoms, the younger family members may respond adequately. This assumption is supported by our finding that second- and third-generation migrants had better stroke knowledge. Given the cultural importance of these more generation families, future studies of these migrant groups may focus on surveying social units such as families rather than individuals.

## Conclusions

To the best of our knowledge, this is the first study of stroke knowledge comparing diabetics with and without migration background. Our data showed good stroke knowledge among fifty percent of native and twenty-two percent of diabetics with migration background. The results were obtained in one community setting and one group of migrants only. Future research about stroke knowledge of diabetics in other migrant populations and community interventions addressing more generation households are necessary. Based on our results and every-day experiences in providing health care to high risk individuals we consider the evaluation of family-centered information strategies a promising approach to improve stroke education.

## Endnote

^a^Official statistical data show that the largest proportion of all migrants to Germany come from Turkey [27]. These include both persons from Turkish and Kurdish origin. We were not able to differentiate these two groups in our survey, so that we are using the terms “patients from Turkey” to describe the country, not heritage of origin.

## Abbreviations

CI: Confidence interval; IQR: Interquartile range; vs.: Versus.

## Competing interests

The authors declare that they have no competing interests.

## Authors’ contributions

BW initiated the study, developed the design, supported the data collection, performed the statistical analysis, and drafted the manuscript. YD participated in the design of the study, performed the data collection, participated in the data analysis and helped to draft the manuscript. SR participated in the data quality control, performed the statistical analysis and helped to draft the manuscript. PB participated in the data collection and helped to draft the manuscript. SG participated in the study design and helped to draft the manuscript. All authors read and approved the final manuscript.

## Pre-publication history

The pre-publication history for this paper can be accessed here:

http://www.biomedcentral.com/1471-2377/13/202/prepub
